# Specialist Savvy Versus Generalist Grit: Elucidating the Trade‐Offs in Adaptive Dietary Ecomorphology Amongst African Green and Bush Snakes

**DOI:** 10.1002/ece3.71414

**Published:** 2025-06-17

**Authors:** Hanlie M. Engelbrecht, Kimberley E. J. Chapelle, Graham J. Alexander

**Affiliations:** ^1^ School of Animal, Plant and Environmental Sciences, University of the Witwatersrand Johannesburg South Africa; ^2^ Department of Anatomical Sciences Stony Brook University Stony Brook New York USA; ^3^ Evolutionary Studies Institute, University of the Witwatersrand Johannesburg South Africa

**Keywords:** graded dietary generalization, inferred adaptation, pulsed evolution, resource partitioning

## Abstract

Kinetic feeding bones of macrostomatan Afrophidian snakes enable them to consume diverse prey types. While significant research has focused on functional feeding morphology in snakes, it often emphasizes broad taxonomic comparisons or species with distinct dietary ecologies. There is limited knowledge of how small variations in prey type composition may influence feeding morphology among closely related species sharing similar ecological niches. African Green and Bush Snakes (*Philothamnus*) feed primarily on frogs (anurophagous) and lizards (saurophagous), but the degree of intraspecific dietary generalization and specialization remains unclear. Thus, our study had three objectives: (1) to evaluate proportional differences in anurophagy and saurophagy between *Philothamnus* species, (2) quantitatively assess the shape differences in four of the main cranial bones functional in feeding, and (3) explore models of diet evolution. We hypothesized that differences in feeding morphology would reflect the extent of dietary specialization and/or generalization. Macroevolution diet analysis results indicated two main diet groups: anurophagous specialists and anuro‐saurophagous generalists. We included 14 species to reconstruct ancestral dietary states within a phylogenetic framework and analysed five representative species using geometric morphometric methods to assess skull shape variation related to dietary specialization and generalization. Geometric morphometric shape analyses show that the jawbones of anurophagous specialists ( *P. angolensis* and *P. hoplogaster* ) have higher mechanical advantage (MA), pronounced posteriorly curved maxillary teeth, deeper mandibular fossae on a more convex shaped compound, wider proximal quadrates, and deeper quadromandibular joint articulations. Conversely, anuro‐saurophagous generalists ( *P. occidentalis* , *P. natalensis* and *P. semivariegatus* ) have longer and thinner jaw bones with lower MA, a more horizontal dorsal quadrate, and high shape variation in maxillae and pterygoids. These findings suggest that dietary morphology is malleable and pervasive even among congeners with fine‐scaled differences in prey type proportions. Dietary specialization and generalizations among the African Green and Bush Snakes appear to exist along a continuum. Dietary ecomorphology appears to be influenced by phylogenetic relationships and may have evolved in dual mode, i.e., gradual specialization and punctuated shifts in dietary generalisation in response to ecological opportunity.

## Graphical abstract

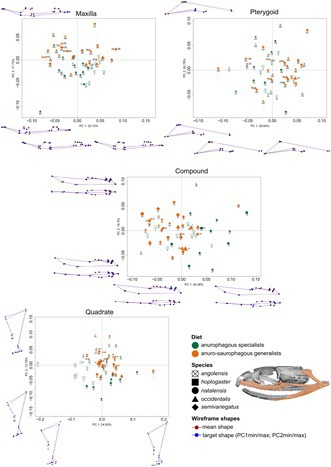

## 1 Introduction

Ecological niches have been shown to have important impacts on the evolution of morphological traits of organisms. For example, Mazel et al. (2017) found that when mammalian species have similar characteristics such as diet, body mass, activity cycles, and foraging strata, it often results in convergent niche evolution. Diet was shown to be an especially important niche dimension of many mammalian species because it is one of the most important resource requirements of animals, especially for endotherms with high rates of energy expenditure. Thus, particular diets can result in the evolution of morphological specializations that promote foraging and feeding efficiency. However, few studies have evaluated the patterns between the evolution of organismal morphology and niche evolution (Ortiz‐Medrano et al. 2016). Such studies have the potential to reveal and explain the functionality of morphological characteristics.

The feeding morphology of snakes represents an interesting case of functional specialization and/or generalization, driven by ecological pressures. Macrostomatan alethinophidian snakes evolved highly kinetic skulls, in that mandibles are loosely connected to the cranium at the back of the skull by means of the quadrate bone. The braincase and nasals of the snake skull are primarily taxonomically informative (Gentilli 2009; Gomes et al. 2020), but may also vary in form such as width and depth to accommodate prey shape (Papežíková et al. 2024). However, the kinetic skull bones including the maxilla, palatopterygoid arch (palatine, pterygoid and ectopterygoid), mandible (dentary and compound) and suspensorium (supratemporal and quadrate) are under direct selection for feeding ecomorphology (Rhoda et al. 2020; Pandelis et al. 2023).

The evolution of increased jaw mobility in many species of macrostomatan snakes allows them to consume relatively large prey items which can exceed 50% of their body mass (Cundall 1983). The anterior tips of the mandibles are connected with a highly stretchable ligament allowing the two mandibles to move independently and to separate widely, greatly increasing the gape and enabling the ingestion of large prey (Gripshover and Jayne 2021). Prey handling and intraoral prey transport are facilitated by the palatomaxillary arch, which includes the pterygoid, ectopterygoid, palatine, and maxilla bones, and can move independently to the rest of the skull (Segall et al. 2023). This allows the head of the snake to advance over the prey (Moon et al. 2019) in what has been termed the ‘pterygoid walk’ (Boltt and Ewer 1964) in species of derived snakes (Segall et al. 2023). The role of the snake maxillae during ingestion appears minimal, whereas a robust dentary and maxilla combination appears to be better suited for grasping and manipulation of prey during capture relative to manipulation (Knox and Jackson 2010). The compound bone serves diverse functions, such as contributing to mechanical advantage for jaw closure during prey capture (Hampton 2011), facilitating the ventromedial movement of the palatopterygoid arch and enabling dorsolateral movement of the dentary tooth row during ingestion (Cundall and Greene 2000).

A wider cephalic condyle could increase the attachment area for mandibular adductor muscles, potentially increasing jaw closure and bite force. Although in vivo measurements of bite force and direct links between tendon attachment area and force output remain largely untested for snakes, studies in other vertebrates have validated model‐predicted bite forces by comparison with actual in vivo measurements (Curtis et al. 2010; Davis et al. 2010; Gröning et al. 2013). Despite some underestimation when factors such as physiological cross‐sectional area, muscle fiber length, and pennation angle are not incorporated, the utility of lever systems and 3D skull models still provides inferential value. Therefore, while caution is warranted, anatomical inferences based on skull lever mechanics and attachment morphology may still offer useful insights into functional adaptation and potential performance trade‐offs across prey types. Higher mechanical advantage could be beneficial for deflating rotund prey such as anurans or crushing hard‐bodied prey such as lizards and snakes. In contrast, lower mechanical advantage may provide faster jaw closing speed, which could be useful for capturing fast, elusive prey such as fish (Alfaro 2002; Vincent et al. 2005; Mori and Vincent 2008; Brecko et al. 2011; Hampton 2011; Rhoda et al. 2020). The quadrate's length and posterior rotation could impose limitations on gape size (Kardong 1977; Johnston 2014; Rhoda et al. 2020), where a larger mouth gape is common for anurophagy as well as piscivory, given the variable shape of fish (Mori and Vincent 2008; Andjelković et al. 2016). Additionally, long mandibular and enlarged posteriorly curved maxillary teeth and/or asymmetrical jaws are regarded as adaptations to grip onto slippery prey in snail and slug‐eating snakes such as species of *Contia*, *Dipsas*, *Pareas*, *Sibynomorphus*, and *Tomodon* (Zweifel 1954; Peters 1960; Bizerra et al. 2005; Chang et al. 2021). Essentially, the skulls of macrostomatan snakes can be highly specialized or generalized to accommodate a wide range of prey types, differing in shape, size, texture, and mobility. This specialization or generalization is reflected in the structure and function of movable elements of the skull, which offer insights into feeding adaptations of advanced snakes, from prey capture and immobilization to prey manipulation, repositioning, and ingestion.

Beyond structural considerations, evolutionary transitions in dietary states provide a framework for understanding how feeding morphology and ecological niches co‐evolve. Ancestral reconstructions of dietary states and analyses of diet evolution can reveal historical patterns underlying present‐day feeding adaptations. To date, studies on snake feeding morphology have primarily focused on higher‐level taxonomic comparisons of functional adaptations. Given the phylogenetic and ecological disparity between snake lineages, significant morphological differences in skull shape with respect to feeding specialization would be expected—largely influenced by phylogenetic inertia (Bellini et al. 2015; Glaudas et al. 2019; Grundler and Rabosky 2021; Maritz et al. 2021). However, intrageneric predator species with similar ecologies are likely to share similar diets and have similar cranial and jaw morphology, rendering it challenging to reliably conclude how dietary ecomorphology is differentiated. Thus, the evolutionary implication of diet niche partitioning has substantial significance, as it indicates the capacity of congeners to exploit different resources, mitigating competition and enhancing ecological coexistence.

Recent studies indicate that fine‐scale variations in feeding kinematics, prey‐type proportions, and prey size can also drive differences in feeding morphology, both amongst snake species at the intrageneric level and between populations of the same species (Heinicke et al. 2020; Chang et al. 2021; Ammresh et al. 2023). Consequently, this presents a promising avenue for comparative studies on structural and functional morphology in snakes, emphasising osteological adaptations in response to fine‐scale differences in dietary niches. Additionally, these comparative studies highlight the evolutionary pathways shaping dietary specialization. Investigating both dietary ecomorphology and the evolutionary trajectories of diet transitions between closely related species with overlapping diets can contribute to a more nuanced understanding of how ecological pressures shaped feeding systems in snakes. The African Green and Bush Snakes, *Philothamnus*, offer a promising model for exploring these relationships—particularly in assessing whether species within the genus exhibit a continuum of dietary specialization or whether all members are true generalists feeding on both frogs and lizards, as currently understood (Loveridge 1958; Broadley 1983; Marais 2004; Spawls et al. 2018).

Distributed across sub‐Saharan Africa, *Philothamnus* species have diverse diets, reportedly preying on frogs, lizards, fish, small birds, rodents, and insects, while frogs and lizards seemingly dominate the diets of *Philothamnus* species (Loveridge 1958; Broadley 1983; Marais 2004; Spawls et al. 2018). However, dietary states for *Philothamnus* species have not yet been formally classified, leaving open questions about whether sympatric or parapatric species exhibit true diet generalization or specialization toward a particular prey type. To address this gap, we investigated how dietary states correlate with feeding morphology and diet evolution within *Philothamnus*, focusing on five representative species for morphology ( *P. angolensis* , *P. hoplogaster* , *P. natalensis* , *P. occidentalis* and *P. semivariegatus* ). We aimed to (1) quantify proportional differences between prey types for the genus, (2) analyse geometric morphometric shape differences in key skull bones amongst five ecologically representative species, and (3) evaluate whether evolutionary models for diet state transitions correspond with diet ecomorphology. We hypothesized that dietary specialization and/or generalization would correspond with distinct morphological adaptations, reflecting evolutionary pressures shaping both feeding systems and dietary niches in this genus.

## 2 Materials and Methods

Cryptic feeding behavior in snakes, seasonal variation in prey availability, the expensive and time‐consuming nature of collecting field data can result in a cumbersome workflow for studies of snake diet and associated skull adaptations. However, recent advances have improved the accuracy of analysing dietary adaptations even amongst closely related lineages with an assumed overlap in the diet niche. Snake diet data, in particular, are now more accessible (Grundler 2020; Maritz and Maritz 2020). Additionally, three‐dimensional micro‐computed tomography (μCT) applications offer non‐destructive and enhanced accuracy to investigate shape variation that could be informative for inferring dietary adaptations within a phylogenetic framework.

### 2.1 Study Taxa and Specimens for Geometric Morphometric Analyses

**FIGURE 1:**
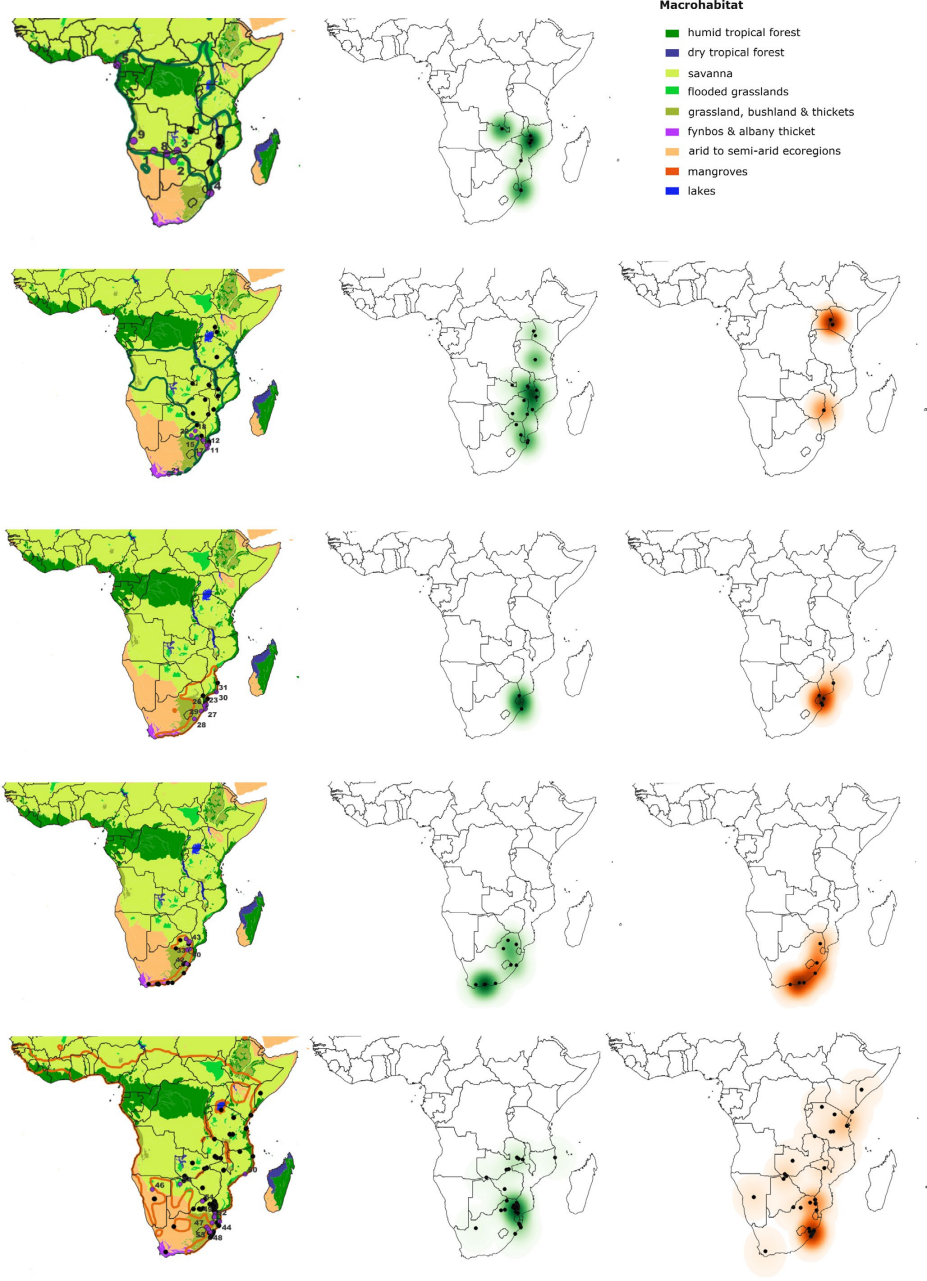
Broad outline of species distributions, macrohabitat affiliations, and geographical localities of specimens used in geometric morphometric analyses (purple symbols), shown in relation to predation records (black symbols) for *P. angolensis* , *P. hoplogaster* , *P. natalensis* , *P. occidentalis* , and *P. semivariegatus* . Kernel Density Estimation (KDE) illustrates the spatial distribution of predation records, with green shades representing areas of anuran predation and orange shades indicating saurian predation. Color intensity reflects predation density, with darker shades indicating higher frequencies of recorded events. Numbers for geographic locations of specimens correspond to specimen numbers in the principal component analysis plots (Figure 3).

To investigate potential morphological links between dietary specialization and/or generalization and feeding apparatus within *Philothamnus*, we focused on five species representing the ecological variation from major phylogenetic clades within the genus (Engelbrecht et al. 2019, 2021). Preserved specimens studied for micro‐computed tomography were from the herpetological collection in the Ditsong National Museum of Natural History, South Africa. The study sample included individuals from across the geographic range of each species, broadly capturing their typical habitat affiliations and overall distributional extent (Figure 1). The sample sizes were: *n* = 10 ( *Philothamnus angolensis* ), *n* = 12 ( *Philothamnus hoplogaster* ), *n* = 9 ( *Philothamnus natalensis* ), *n* = 12 (*Philothamnus occidentalis*), and *n* = 11 (*Philothamnus s. semivariegatus*), primarily the northeastern lineage in South Africa; *P. s. semivariegatus* 2, as per Engelbrecht et al. (2019) for micro‐computed tomography. We focused on adult specimens to investigate shape variation that is independent of age‐related changes in cranial morphology (Appendix A).

### 2.2 Dietary States

Diet records were retrieved from publicly available databases via: (1) the R package SquamataBase literature‐based v.4.0.0 (Grundler 2020) and (2) citizen science using social media platforms (Google Images, iNaturalist and a Facebook group: Predation Records—Reptiles and Frogs (Sub‐Saharan Africa), as well as historical literature records (Maritz and Maritz 2020). A total of 243 predation records were captured, which included both direct observations and dissections for gut content (Dryad, DOI: https://doi.org/10.5061/dryad.2jm63xt0g), ranging from 9 to 128 observations per each of the five focal species (Table S1, https://doi.org/10.5061/dryad.2jm63xt0g). Predation records included individuals from across the geographic range of each species, broadly capturing their typical habitat affiliations and overall distributional extent (Figure 1). To place the five focal species in a phylogenetic framework, additional diet records were collected for a total of 14 *Philothamnus* species and three outgroup taxa; *Dispholidus typus* , *Hapsidophrys lineatus* and *Thelotornis capensis* . We tested for differentiation in dietary states using a continuous‐time Markov chain process within a Dirichlet‐multinomial framework in the Macroevolution R package v.4.0.1. (Grundler and Rabosky 2020). The latter not only models the evolution of diet niche partitioning within a phylogenetic context but is also advantageous in allowing for the modelling of a multifaceted diet per predator species. It also accounts for uncertainty in terminal taxa diet state assignments arising from variations in sampling intensity. Retrieved diet records were taxonomically categorized per prey species as, “birds”, “frogs”, “lizards” and “small mammals” and other). A previously published phylogeny was used in association with diet data collected in this study (Engelbrecht et al. 2019, 2021) to evaluate diet niche partitioning amongst study taxa, using model parameters as per Grundler and Rabosky (2020). We ran the model for 100,000 iterations, sampling every 100th iteration with a 10% burn‐in. Predator species were considered diet specialists if the prey category represented ≥ 75% of recorded prey.

We used Kernel Density Estimation (KDE) mapping in QGIS v 3.383 (QGIS Development Team 2024) to identify spatial hotspots of predation events, separately for frog and lizard prey, across all predator species. The KDE is a non‐parametric spatial analysis technique that estimates the probability density function of point events over a continuous space. In this context, it helps reveal regions where frog and lizard predation events are most densely clustered, thereby highlighting potential zones of resource‐use concentration by each predator species.

### 2.3 Micro‐Computed Tomography

Specimens were scanned at the Wits Microfocus X‐ray computed tomography (micro‐CT) facility of the Evolutionary Studies Institute (ESI) at the University of the Witwatersrand, Johannesburg, South Africa, which uses a Nikon Metrology XTH 225/320 LC dual source industrial CT system, and at the Micro‐focus X‐ray tomography facility (MIXRAD) at the South African Nuclear Energy Corporation (NECSA). The following CT‐scanning settings were used: 70 kV, 120 μA, 1fps, and the resulting average voxel size was 1 × 1 × 1 = 0.01605 mm. Three‐dimensional surface models of skulls were reconstructed in VGSTUDIO MAX v3.2 (Volume Graphics GmbH, Germany), using a 30–40 μm resolution. Movable elements (compound, maxilla, pterygoid, and quadrate) on the left side of each skull were segmented separately (Dryad, DOI: https://doi.org/10.5061/dryad.2jm63xt0g), and landmarked using Amira version 5.4.5 (Stalling et al. 2005).

### 2.4 Landmark Scoring, Shape Variables, and Mechanical Advantage

Landmarks were adapted from Andjelković et al. (2016): 14 compound, 14 maxilla, 8 pterygoid, and 9 quadrate. Landmark definitions are available on Dryad (DOI: https://doi.org/10.5061/dryad.2jm63xt0g). While we acknowledge that other cranial elements also contribute to feeding mechanics, we selected these four bones because they represent key functional modules with established biomechanical roles across diverse feeding strategies (Gans 1961; Rhoda et al. 2020). Landmarks were placed in Amira 5.4.5 and subsequently analysed (Procrustes alignment and principal component analysis [PCA]) in RStudio 2022.07.2 using the geomorph package 4.0.4 (Baken et al. 2021; Adams et al. 2023). Mechanical advantage was calculated by dividing the in‐lever length by the out‐lever length. In‐lever length (quadromandibular joint to mandibular fossa) and the out‐lever length (total mandible length) were measured as straight‐line distances following Vincent et al. (2007) and Hampton (2011). This two‐dimensional lever‐based approach is widely used in vertebrate ecomorphology and biomechanics (Herrel and Aerts 2004), and studies in lizards and other squamates have shown that such morphological proxies correlate with in vivo bite force performance (e.g., Curtis et al. 2010; Gröning et al. 2013). While 2D lever models may underestimate bite force compared to more detailed 3D models—particularly due to anatomical simplifications (Davis et al. 2010)—they still perform well in predicting general trends and are commonly applied where direct measurements are unavailable. Additionally, bite force predictions in squamates are often underestimated due to high muscle pennation, with recommended adjustments of up to three times the predicted value to account for these inaccuracies. Bite forces also increase and joint forces decrease as the bite point shifts posteriorly along the jaw, with the most posterior bite location generating a bite force almost double that of the most anterior bite (Curtis et al. 2010). In this study, our aim was not to estimate absolute bite force values, but rather to examine relative differences in mechanical advantage across species with differing dietary specializations. As such, our use of standardized lever‐based metrics provides a biologically meaningful framework to infer interspecific comparisons of MA, even if it does not fully capture the complexity of dynamic bite function in snakes.

### 2.5 Relationships Between Size, Shape, Diet, and Mechanical Advantage

For each cranial element, we tested for evolutionary allometry by looking at shape against size (centroid size [CS]) by performing a phylogenetic Procrustes regression (Adams 2014) with the function procD.pgls (10,000 iterations; Mitteroecker et al. 2004) from the geomorph package 4.0.4 in RStudio 2022.07.2. We also tested the hypotheses that dietary specializations influence cranial element shapes and mechanical advantage. This was done through a phylogenetic Procrustes regression (Adams 2014) with the geomorph function procD.pgls (10,000 iterations; Mitteroecker et al. 2004). Due to significant allometry, we also used allometry‐free shape in a second set of subsequent analyses by computing the shape residuals extracted from a phylogenetic Procrustes ANOVA (Goodall 1991) of cranial element shape against size. Sampling per sex per species was not adequate for statistical analyses of sexual dimorphism in bone shape; however, we were cognizant that the five focal *Philothamnus* species are sexually dimorphic, with females having higher ventral scale counts and fewer subcaudal scales than males (Hughes 1985), thus we considered this a potential confounding factor in our interpretation of shape variation for the four skull bones studied.

### 2.6 Evolutionary Transitions in Dietary States

To complement our morphometric findings and assess whether dietary specialization or generalization evolved in a phylogenetically patterned manner, we conducted a macroevolutionary analysis of diet across a previously published *Philothamnus* phylogeny (Engelbrecht et al. 2019, 2021). We reconstructed ancestral dietary states with stochastic character mapping via the plotSimmap function in the Phytools R package (Revell 2012). This allowed us to visualize transitions in diet type—specifically, shifts between anurophagy, saurophagy, or mixed diets—through evolutionary time. To assess the degree of phylogenetic signal in diet, we calculated Blomberg's *K* and Pagel's *λ* using the phylosig function in Phytools (Blomberg et al. 2003; Pagel 1999). A *K* > 1 or *λ* near 1 indicates that closely related species exhibit more similar diets than expected under Brownian motion. The ancestral diet state reconstruction was superimposed on a density plot for diet trait value distribution, which was generated using the geom_density function in the ggplot2 R package (Wickham 2016). To further explore the tempo and mode of dietary evolution, we compared evolutionary models for dietary specialization and/or generalization within the genus by using a restricted maximum‐likelihood estimation (REML) for fitting Lévy processes as implemented in the R package pulsR (Landis and Schraiber 2017). We specifically compared the statistical fit of incremental change, incremental stationarity, explosive change, and pulsed evolution, respectively Brownian motion (BM), Ornstein‐Uhlenbeck (OU), early‐burst (EB), jump normal (JN), normal inverse Gaussian (NIG), Brownian + jump processes (BMJN and BMNIG). Akaike Information Criterion corrected for small sample sizes (AIC_c_) and associated log‐likelihood scores (InL) were used to assess relative model support by comparing weighted AIC_c_ scores.

## 3 Results

### 3.1 Dietary States

**FIGURE 2:**
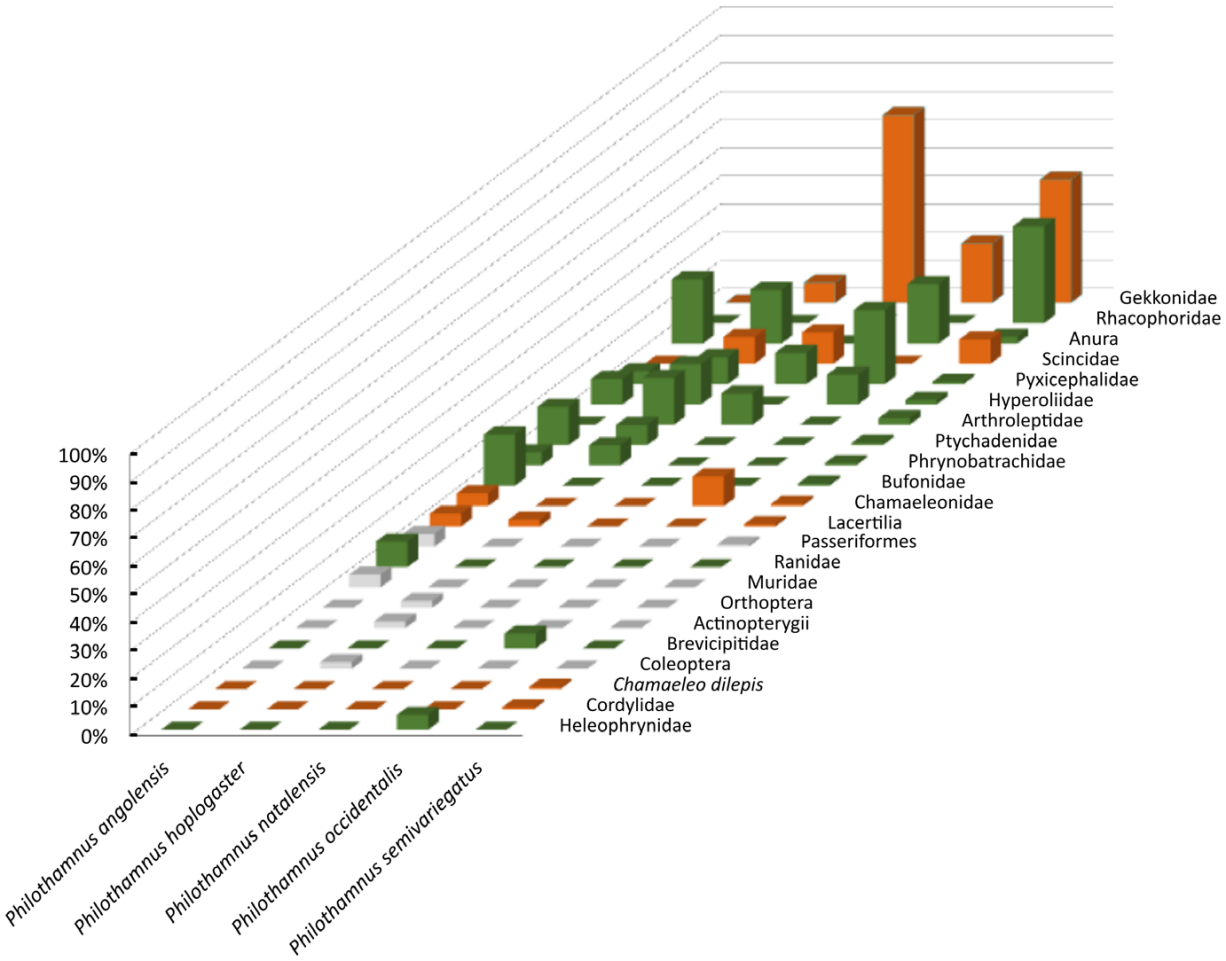
Diet composition for the five focal *Philothamnus* species studied for geometric morphometrics. The prey type composition for specialist predators; *P. angolensis* and *P. hoplogaster* constitutes 97% anurans. In contrast, the diet of generalists; *P. occidentalis* , *P. natalensis* , and *P. semivariegatus* comprise 44% anurophagy and 56% saurophagy.

At least two dietary states were evident amongst the *Philothamnus* species included in the Macroevolution diet niche partitioning analysis. *Philothamnus angolensis* and *P. hoplogaster* are the only dietary specialists feeding almost exclusively on anurans (97%) relative to lizards. *Philothamnus natalensis* , *P. occidentalis* , and *P. semivariegatus* feed on lizards (56%) and frogs (44%) with almost equal frequency (Figures 1 and 2). Species show varying levels of dietary diversity. Amongst the five focal species, *P. hoplogaster* has the most diverse anuran diet, while *P. occidentalis* and *P. semivariegatus* have the most diverse anuro‐saurophagous diets. For both diet groups, geckos constitute the bulk of lizard prey species relative to skinks and chameleons.

The KDE mapping reveals that generalist species— *P. natalensis* , *P. semivariegatus* , and *P. occidentalis* —show broad overlap in both frog and lizard predation zones, suggesting flexible dietary strategies across shared habitats. In contrast, the specialist species— *P. hoplogaster* and *P. angolensis* —display hotspots that are primarily dominated by frog predation events in close geographical proximity to the lizard predation zones of generalist species. The latter supports anurophagous preference and specialization for *P. hoplogaster* and *P. angolensis* . This spatial segregation of prey use also aligns with ecological resource partitioning and suggests niche differentiation driven by prey specialization in the presence of generalist species.

### 3.2 Shape Variation of Skull Elements and Mechanical Advantage

Results for Procrustes analyses indicate a significant relationship between shape variation and centroid size, both with and without correction for phylogeny (Table 1), suggesting that changes in centroid size are associated with measurable differences in shape for each of the skull elements (compound, maxilla, pterygoid, and quadrate) studied. Additionally, a significant relationship was found between shape variation and diet type after accounting for allometry, indicating that diet plays an important role in shaping variation in the four bones studied, regardless of known sexual dimorphism. Mechanical advantage (MA) is also significantly higher in anurophagous specialists (0.31–1.11) than for the anuro‐saurophagous generalists (0.27). However, it should be considered that the geometric morphometric shapes of the four movable skull elements and mechanical advantage investigated were not significantly different between the two snake diet groups when corrected for phylogeny.

**TABLE 1:** Procrustes ANOVA/regression (procD.lm) and phylogenetic ANOVA/regression (procD.pgls) model results for Procrustes aligned shape variables (coordinates), centroid size (CS), diet group (anurophagous specialists *P. angolensis* and *P. hoplogaster* ; and anuro‐saurophagous generalists *P. natalensis* , *P. occidentalis* , and *P. semivariegatus* ), and mechanical advantage (MA).

Bone	DF	SS	MS	Rsq	*F*	*Z*	Pr(>*F*)
Model: procD.lm coords ~ CS							
Compound	1	0.0270	0.027000	0.08091	4.5777	3.0344	0.001**
Pterygoid	1	0.015649	0.015649	0.05662	3.1211	2.3544	0.009*
Quadrate	1	0.04399	0.043988	0.06716	3.7437	3.193	0.001**
Maxilla	1	0.023657	0.0236572	0.08092	4.578	3.2302	0.001**
Model: procD.pgls coords ~ CS							
Compound	1	0.012083	0.0120832	0.05142	2.8191	2.1268	0.021*
Pterygoid	1	0.016987	0.0169868	0.07179	4.0216	2.6926	0.004**
Quadrate	1	0.03462	0.034616	0.06974	3.8983	3.4487	0.001**
Maxilla	1	0.01234	0.0123398	0.06234	3.4573	2.8903	0.004**
Model: procD.lm coordinates ~ diet group							
Compound	1	0.04408	0.044082	0.1321	7.9148	3.7779	0.001**
Pterygoid	1	0.014155	0.0141547	0.05121	2.8069	2.1311	0.017*
Quadrate	1	0.04451	0.044513	0.06796	3.7917	3.3356	0.002**
Maxilla	1	0.02926	0.0292604	0.10008	5.7829	3.8594	0.001**
Model: procD.pgls coordinates ~ diet group							
Compound	1	0.000337	0.0003371	0.00143	0.0747	−4.4022	1
Pterygoid	1	0.000211	0.0002108	0.00089	0.0464	−3.444	1
Quadrate	1	0.00048	0.0004821	0.00097	0.0506	−6.8689	1
Maxilla	1	0.000439	0.0004387	0.00222	0.1155	−4.9506	1
Model: procD.lm allometry free coordinates ~ diet group							
Compound	1	0.036018	0.036018	0.11744	6.9193	3.1791	0.001**
Pterygoid	1	0.012176	0.0121758	0.0467	2.5473	1.9888	0.023*
Quadrate	1	0.04982	0.049825	0.08155	4.617	4.1401	0.001**
Maxilla	1	0.028373	0.0283732	0.10559	6.1389	3.532	0.001**
Model: procD.lm MA ~ diet group							
	1	0.9651	0.96506	0.0886	5.0552	1.8795	0.023*

*Note:* Significant differences are indicated by a *p < 0.05 and **p < 0.005.

**FIGURE 3:**
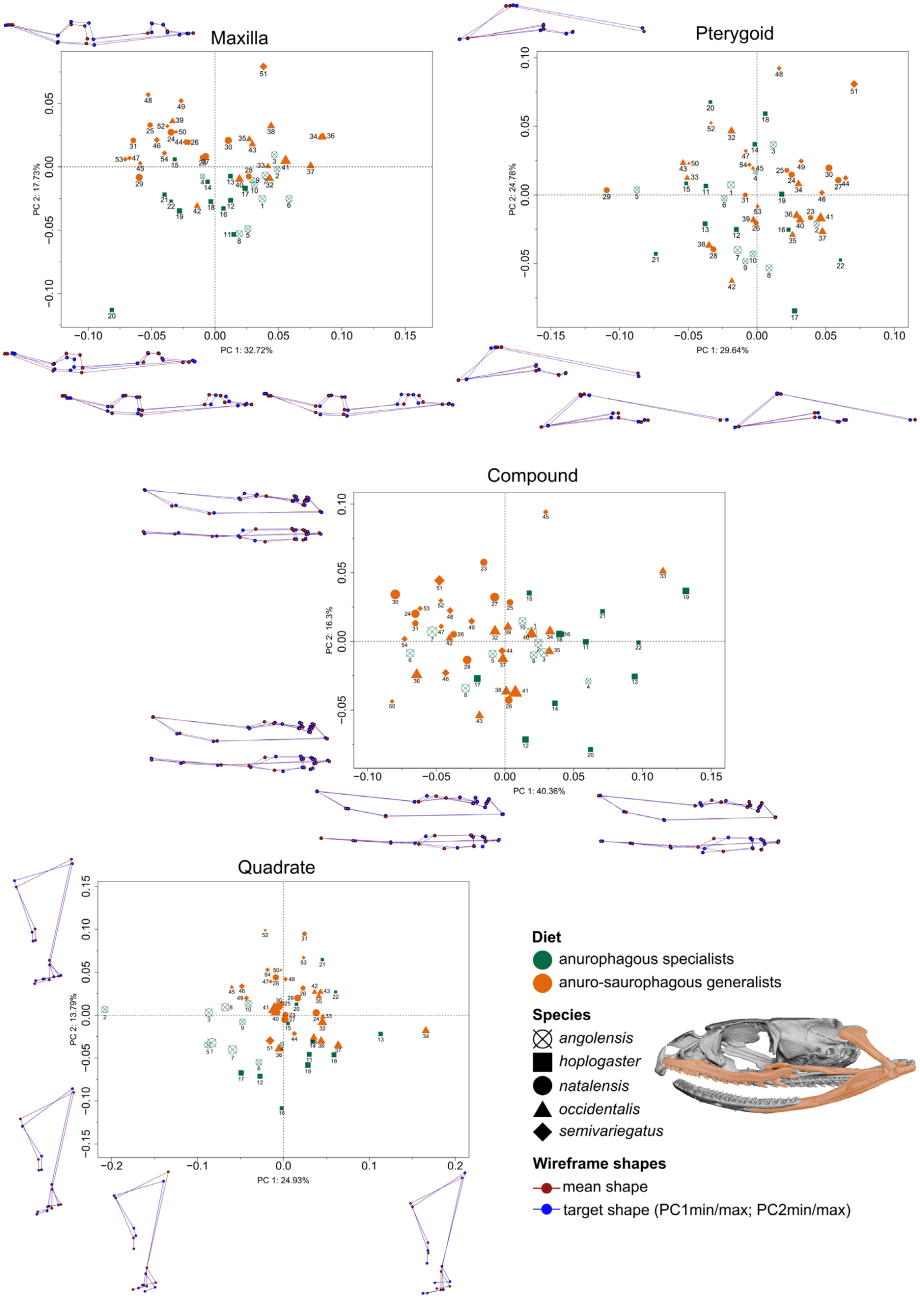
Results of the principal component analysis for three‐dimensional bone shapes, between anurophagous specialists *P. angolensis* and *P. hoplogaster* and anuro‐saurophagous generalists *P. natalensis* , *P. occidentalis* and *P. semivariegatus* . Wireframes show shape changes relative to extremes of variation along each principal component axis. Data points are scaled relative to centroid size and numbered according to the geographical locations for specimens used in the geometric morphometric analyses (Figure 1).

**FIGURE A1:**
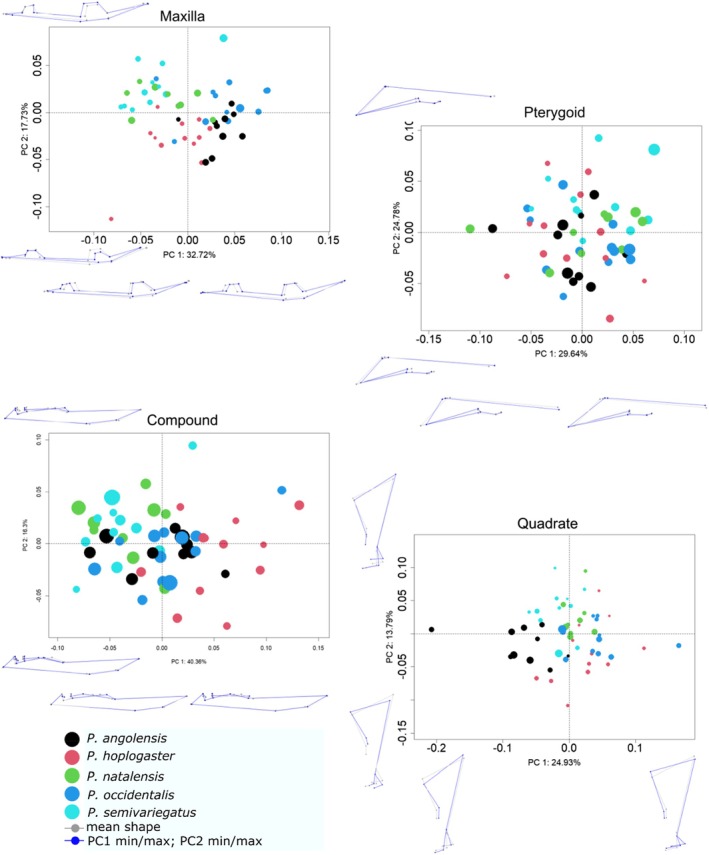
Results of the principal component analysis (PC1 and PC2) for three‐dimensional bone shapes by species. Wireframes show shape changes relative to extremes of variation along each PC axis.

**FIGURE A2:**
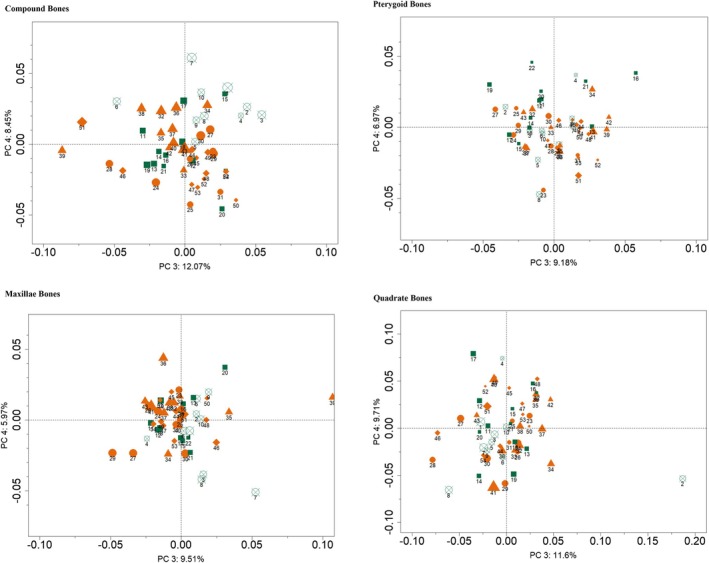
Results of the principal component analysis (PC3 and PC4) for three‐dimensional bone shapes by species. Wireframes show shape changes relative to extremes of variation along each PC axis. Please refer to the main Figure 3 legend for symbol interpretation.

Geometric morphometric shape variations of the four bones are separated on two extremes of the three‐dimensional morphological axis (Figure 3; Appendix A: Figures A1 and A2). Principal component 1 described most of the bone shape variation between diet groups (24.93%–40.36%). However, the PC1 and PC2 contributions to bone shape variation between diet groups may be confounded by *P. occidentalis* as it consistently falls within the intermediate morphospace relative to anurophagous specialists ( *P. angolensis* and *P. hoplogaster* ) and anurophagous‐saurophagous generalists ( *P. natalensis* and *P. semivariegatus* ) (Appendices A. The maxilla is the most morphologically distinct between the two diet groups, followed by the quadrate and the compound bones, while the pterygoid shows high intraspecific shape variation for the anuro‐saurophagous generalists. Please refer to Appendix B for detailed shape variation across PC1 min–max and PC2 min–max for each of the four bones studied.

#### 3.2.1 Maxilla

Anuro‐saurophagous generalists have shorter maxillae than the anurophagous specialists. This is especially true for *P. natalensis* and *P. semivariegatus* (Figure 3; Appendix A). These species also have a longer interspace between the palatine‐ectopterygoid facets, and this bar is narrower relative to anurophagous specialist *P. angolensis* as well as *P. occidentalis* . Anurophagous specialists have overall smaller palatine facets but a broader base for the ectopterygoid processes. The maxillary teeth of anurophagous specialists show a more pronounced curvature backwards, and posterior teeth resemble fangs, enlarged and thicker compared to thinner, more needle‐like posterior maxillary teeth in *P. natalensis* and *P. semivariegatus* . All species appear to have cutting edges on maxillary teeth that are more prominent on enlarged posterior teeth. A posterior diastema is not evident in any of the species studied. The total shape variation between the five species along PC1 is 32.72% and corresponds to the relative positions of facets, the width of the bar between the palatine‐ectopterygoid facets, and the length of the maxillae.

Principal component 2 accounts for 17.73% of total variation between the five species and corresponds mostly to the variation in the shape of the palatine and ectopterygoid processes, with *P. occidentalis* showing intermediate morphological shapes for these traits (Appendix A).

#### 3.2.2 Compound

Anurophagous specialists have relatively shorter and thicker compounds that show a more dorsal convexity anteriorly. The articulation with the quadrate is also deeper in anurophagous specialists. For anuro‐saurophagous generalists, the compound articulation with the dentary (intermandibular hinge) appears more extensive, and the mandibular fossa and surangular are more superficial and elongated. Principal Component 1 accounts for 40.36% of the total shape variation between the five species and represents the variation in the mandibular fossa and surangular crest.

The total shape variation between the five species along PC2 is 16.3%, which also represents the variation in the mandibular fossa and the surangular crest, in addition to the relative position of the saddle‐shaped articular surface, the total length of the compound bone, and the size of the intramandibular hinge.

#### 3.2.3 Quadrate

In anurophagous specialists, the quadrate shows extensive articulation with the compound. The stylohyal process is also dorsoventrally higher. Overall, the width of the dorsal margin of the quadrate varies between species and diet groups; however, the dorsal margin is continuously sloping in *P. occidentalis* and anurophagous specialist *P. hoplogaster* , and more horizontal in anuro‐saurophagous generalists, while angular (horizontal with a sloping posterior extension) in *P. angolensis* . Principal component 1 accounts for 24.93% of the total variation between the five species and illustrates multiple aspects of shape variation, including the dorsalmost margin of the quadrate, positions of the stylohyal process, dorsal extent of the ventral fossa, and the lateral extension of the condyle.

Principal component 2 explains 13.79% of the differences observed between the five species and also illustrates the variation in the dorsal margin of the quadrate, as well as the extent of the ventral fossa, along with the shape of the stylohyal process.

#### 3.2.4 Pterygoid

The shape of the pterygoid is more variable amongst anuro‐saurophagous generalists. Overall, total pterygoid length between the two diet groups appears the same; however, the length of the anterior compared to the posterior parts of the pterygoid differs between the two diet groups. Anurophagous specialists have a relatively longer and narrower pterygoid anteriorly and laterally extended pterygoids. The ectopterygoid process on the pterygoid is more ventrally and posteriorly positioned in anurophagous specialists.

Principal Component 1 contributes 29.64% of the total variability among the five species and represents the variation in the position of the tooth row, the position of the ectopterygoid process on the pterygoid, and the positions of the posterodorsal most point of the pterygoid. Principal Component 2 captures 24.78% of the total variation among the five species and represents variation in the mediolateral extension of the posterior part of the pterygoid as well as the width of the anterior part that contains the tooth row.

### 3.3 Dietary State Evolution

The phylogenetic signal in dietary states, as measured by Blomberg's *K*, was 2.24, indicating a strong phylogenetic signal for *Philothamnus* diet evolution. Additionally, Pagel's lambda was 1.05, also suggesting that diet evolution is highly influenced by phylogenetic relationships, with near‐perfect phylogenetic signal. These results collectively highlight the significant role of phylogeny in shaping dietary traits within the genus.

**FIGURE 4:**
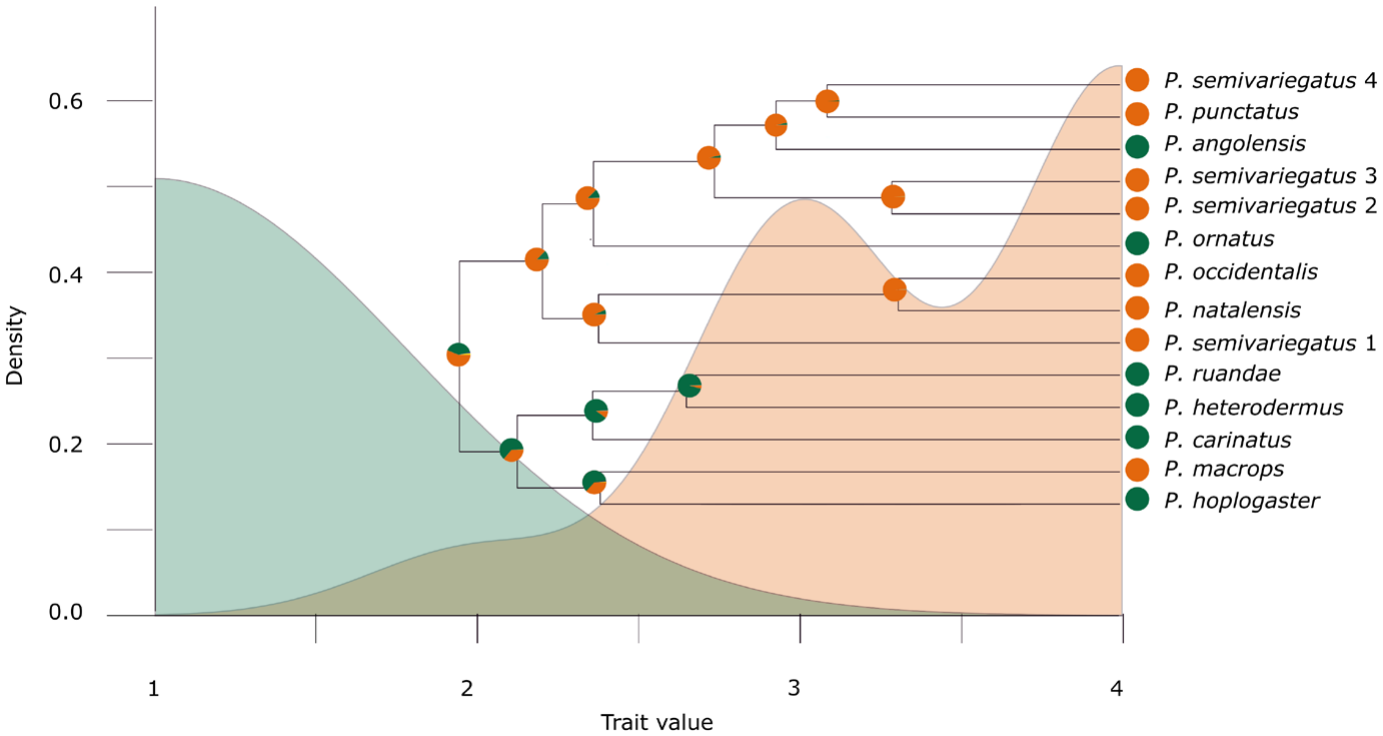
Dietary evolution of *Philothamnus*. Phylogeny, ancestral diet state reconstruction, and dietary trait distribution for 14 selected *Philothamnus* species including the five focal species studied for geometric morphometric analysis. Ancestral dietary state reconstruction included taxa from sister dispholidine genera to polarize dietary states within *Philothamnus*. The ancestral diet reconstruction overlays the trait distribution (density curves) for taxa categorized by dietary state: Anurophagous specialists (green) and anuro‐saurophagous generalists (orange). Overlapping areas between the green and orange curves present shared trait values, suggesting intermediate or transitional states that blur the boundaries between dietary specialization and generalization. Peaks in trait distributions at 1 (anurophagous specialization), 3, and 4 (anuro‐saurophagous generalization) reflect divergent evolutionary trajectories within the genus.

The strong phylogenetic signal suggests dietary evolution is tightly linked to lineage history, while ancestral diet state reconstruction indicates two distinct evolutionary trajectories: one toward anurophagy specialization and the other toward a generalist anuro‐saurophagous diet. However, the best‐supported evolutionary model (BMJN) indicates that while some changes were gradual, others occurred in sudden jumps, consistent with pulsed evolution (Figure 4). The AIC_c_ model comparison identified BMJN as the best‐fitting model, with a Delta AIC_c_ of 0 and a weight approximating 1, providing strong support for a dual‐mode evolutionary dynamic, i.e., dietary specialization and generalization within the genus (Appendix C). Other models, such as BM and BMNIG, exhibited high Delta AIC_c_ values (60–77), indicating poor support relative to BMJN. The combination of Brownian motion (BM) with Jump Normal (JN) in the BMJN model captures the two distinct patterns of evolutionary change that may have occurred simultaneously. The Brownian motion (BM) component describes gradual, incremental changes consistent with random drift or weak selection. In contrast, the JN component captures punctuated, explosive shifts in dietary traits, likely driven by strong selection pressures or environmental events. The observed heavy‐tailed and negatively skewed distribution for anurophagy deviates from the standard assumptions of BM (Figure 4), which typically predicts a symmetric, Gaussian distribution. However, the anurophagy trait distribution pattern suggests selection toward lower trait values and a narrow trait distribution range, which instead reflects stabilizing selection for specialization. In contrast, the generalist clade demonstrates greater trait diversity, consistent with adaptive diversification. Multimodality for anuro‐saurophagous generalists suggests that trait values are not centered around a single value but are instead distributed across distinct clusters. Multiple peaks could also reflect dietary specialization and/generalization evolving from different ancestral states. The rapid shifts observed in dietary states, from the ancestral diet state to distinct trait distribution peaks (3 and 4), align with the concept of pulsed evolution, indicating bursts of adaptive change.

## 4 Discussion

Comparative studies of snake dietary ecomorphology frequently examine distantly related taxa, highlighting traits that enhance the feeding efficiency of prey with varied characteristics, including size, shape, texture, and mobility. Our study, however, focused on closely related snake predator species with substantial overlap in diet, recognizing at least two dietary states among five of at least 28 species of African Green and Bush Snakes (Engelbrecht et al. 2019; Trape et al. 2021; Greenbaum et al. 2023); anurophagous specialists ( *P. angolensis* and *P. hoplogaster* ) and anuro‐saurophagous generalists (*P. natalensis, P. occidentalis* and *P. semivariegatus* ). Accompanying geometric morphometric shape variations indicate that the two predator groups are morphologically distinct, each with unique features to enhance prey capture and retention. The two dietary groups also show differences in mechanical advantage, mouth gape, and morphological traits that could facilitate intraoral prey transport. By examining only four kinetic skull bones among *Philothamnus* species, we showed that feeding morphology in snakes could be highly malleable. We highlight how congeners may have evolved along two trajectories along a fine‐scaled generalist‐specialist dietary continuum, adapting to subtle variations in the proportional composition of the same prey types.

### 4.1 Morphological Specialization for Anurophagy

Jawbones of *Philothamnus angolensis* and *P. hoplogaster* have distinct osteological shapes indicating tailored adaptations for anurophagy relative to those of anuro‐saurophagous generalists. Both species have higher mechanical advantage (MA) and pronounced posteriorly curved maxillary teeth, which are common traits for snakes predating on bulky and slippery prey such as anurans. These morphological features are inferred to generally aid in immobilising and gripping onto prey (Wainwright and Richard 1995; Knox and Jackson 2010; Hampton 2011; Andjelković et al. 2016). Differences in maxillae dentition further support our findings of anurophagous specialization relative to anuro‐saurophagous generalization. The higher number of maxillary tooth sockets for *P. angolensis* , *P. hoplogaster* , and *P. occidentalis* (respectively 23–31, 21–33 and 19–29) compared to *P. natalensis* and *P. semivariegatus sensu lato* (respectively 18–23 and 17–25) (Hughes 1985) suggests greater functionality for gripping onto slippery prey (Knox and Jackson 2010). Deeper mandibular fossae on the compound and wider proximal quadrates may strengthen the support of jaw adductor muscles (Cundall 1995; Johnston 2014), which could in turn increase bite force and contribute to the effective immobilization of bulky prey.

The convex shape of the compound bone may serve a unique function for effectively feeding on slippery prey, such as anurans. A bow‐shaped compound bone is common amongst snake species consuming slippery and/or elongated prey types (Rhoda et al. 2020; Pandelis et al. 2023). For example, *Cylindrophis ruffus* feeds on snakes, caecilians, and eels (Kupfer et al. 2003), *Atractus* and *Geophis* species primarily feed on annelids, *Dipsadini* are snail‐eating snakes (Greene 1983; Balestrin et al. 2007; Zaher et al. 2014) and *Xenodon*, which preys on anurans (Vitt 1983), all have pronounced convex‐shaped compound bones. Given that slippery prey present retention challenges for snakes, a bow‐shaped compound may serve a cupping function to prevent prey escape. Overall, the relatively higher mechanical advantage (MA), distinct posterior maxillary teeth, and features of the quadrate and compound bones may enhance the ability of *P. angolensis* and *P. hoplogaster* to capture, immobilize, and retain slippery anurans more effectively than generalist *Philothamnus* predators.

For *Philothamnus angolensis* and *P. hoplogaster* , the deeper articulation of the quadrate with the compound may stabilize the saddle quadromandibular joint, which is necessary for handling recalcitrant prey such as anurans (Mori and Vincent 2008; Hampton 2011). Given that the mediolateral distance between quadratomandibular joints generally limits prey diameter (Gans 1961), this deeper articulation at these joints may allow for greater lateral distension in anurophagous specialists. In combination, the inferred greater lateral distension, stability of the quadratomandibular joints, and mediolateral extended pterygoids may enable the consumption of wider prey, such as anurans compared to lizards. Deeper articulation of the quadratomandibular joints may also facilitate wider abduction (i.e., mouth gape), another common adaptation for anurophagy specialization in snakes (Mori and Vincent 2008; Vincent et al. 2007). At the same time, an increased posterior rotation inferred from the shape of the dorsal margin of the quadrate in anuran specialists may cause ventral displacement of the palatomaxillary arch during protraction of the pterygoid walk (Kardong 1977). In turn, ventral displacement of the palatomaxillary arch could mitigate gravitational forces when swallowing heavier, slippery anurans during intraoral movement. Thus, shape variables in the feeding bones of anurophagous specialists appear well‐suited to facilitate prey capture, retention, and intraoral transport, enabling the swallowing of bulky, slippery anurans. These inferred morphological adaptations may allow *P. angolensis* and *P. hoplogaster* to outcompete generalist predators in anurophagy.

### 4.2 Potential Morphological Trade‐Offs for Anuro‐Saurophagous Generalization

Compared to anurophagous specialists, anuro‐saurophagous generalists, *P. occidentalis* , and especially *P. natalensis* and *P. semivariegatus* have longer, thinner jaw elements, which are associated with a lower MA. Slender jaw features in snakes typically enhance speed and agility during prey capture. Given that microhabitat use is known to influence predation kinematics for snakes (Vincent et al. 2005; Segall et al. 2016, 2019; Pandelis et al. 2023), and *Philothamnus* species are diurnal active foragers, the high mobility of lizards in cluttered vegetation within arboreal microhabitats may drive the morphology that enhances predation speed and agility, especially when considering the general bioenergetics of active foragers (McLaughin 1989; Beaupré and Montgomery 2007). The horizontal dorsal margin of the quadrate observed for generalists is inferred to limit posterior rotation of the quadrate relative to the supratemporal (Kardong 1977; Rhoda et al. 2020), potentially resulting in a smaller mouth gape, which could further favor handling of flatter prey shapes, such as lizards. Thus, the morphological shape of jaw elements in dietary generalists may be better suited for capturing elusive prey such as faster‐moving, flatter, and elongated geckos and skinks, without excluding anurans.

In contrast, high shape variation of maxillae and pterygoids in anuro‐saurophagous generalists suggests versatile kinematics for capturing and intraoral prey transport. Additionally, shallower compound and quadrate articulations may enhance flexibility in the handling of varying prey shapes, i.e., anurans and lizards. Thus, the feeding morphology of dietary generalists in *Philothamnus* likely represents a functional balance between speed and flexibility, with capturing speed being effective for preying on elusive lizards and handling flexibility advantageous for manipulating bulky anurans. The inferred functional balance between speed and flexibility exemplifies a typical generalist strategy: “jack of all trades, master of none” (Pianka 1978; Huey and Hertz 1984). This strategy typically emerges when the relevant trade‐off is weak and environmental heterogeneity is high (Débarre and Gandon 2011). For dietary generalists this may reflect relatively equal access to both lizard and anuran prey when foraging across heterogeneous microhabitats, from cluttered vegetation to features like rock crevices.

Our findings for *Philothamnus* fine‐scaled differences in feeding morphology linked to variation in dietary states of the same prey types suggest that dietary morphology in snakes may be under extreme selection pressure and/or exhibit phenotypic plasticity. Supporting this idea, recent studies have shown that snake species have similar degrees of morphological adaptations related to fine‐scaled differences in diet. For example, *Pareas* species have asymmetrical jaws associated with varying proportions of snails and slugs, while populations of *Notechis scutatus* display phenotypic plasticity in jaw size relative to prey size (Chang et al. 2021; Ammresh et al. 2023). Consequently, these adaptations in dietary morphology, particularly with respect to proportional differences of the same prey types among closely related taxa, may be pervasive across snake lineages.

### 4.3 Evolutionary Implications of Dietary Specialization and Generalization Within *Philothamnus*

Evolutionary patterns of reconstructed dietary states and diet trait distributions suggest that anurophagous specialization was advantageous for species that persisted in forest habitats since the Early Miocene. In contrast, the pulsed evolution of anuro‐saurophagous generalization is likely a result of radiation into relatively novel and diverse ecological and dietary niches. The pattern of diet evolution is associated with multiple species divergence events linked to the emergence and expansion of open habitats in sub‐Saharan Africa (Engelbrecht et al. 2021). The latter likely provided ecological opportunities for adaptations in dietary ecomorphology. The observed phylogenetic signal may additionally reflect the evolutionary history of the genus, with closely related forest specialists exhibiting anurophagous specialization and closely related habitat generalists showing anuro‐saurophagous generalization. Morphological adaptations supporting dietary specialization and generalization suggest an adaptive radiation for African Green and Bush Snakes since the Early Miocene, resulting in divergent dietary niches.

Graded ecomorphological specialization‐generalization appears to be present amongst species studied. Diet diversity appears to be correlated with the level of habitat specialization or generalization, which is also reflected in dietary morphology. For example, amongst the three generalist species, *P. natalensis* , *P. occidentalis* , and *P. semivariegatus* , *P. occidentalis* may represent a third dietary ecomorph with a higher proportion of anurophagy, but not quite at the specialized level of *P. angolensis* and *P. hoplogaster* . The latter exemplifies the continuum of resource specialization and generalization observed for many taxa (Poisot et al. 2015). The specialist‐generalist ecological continuum typically arises from resource partitioning, where each species causes one of its predecessors to relinquish a portion of its resource allocation. As a result, resource gradients become progressively smaller segments as the number of species increases over evolutionary time (Schluter 2000a, 2000b). Consequently, dietary specialization and generalization within the genus are likely shaped by spatiotemporal factors.

Over evolutionary time, homogenous environments typically give rise to resource specialists, while heterogeneous conditions can promote resource generalization (Débarre and Gandon 2010). However, for *Philothamnus* species, sympatric and parapatric distributions across heterogeneous environments likely drive diet specialization and generalization linked to differences in microhabitat use, thereby reducing ecological competition. For example, *P. angolensis* is patchily distributed across coastal forests, moist savannas, and arid savanna margins, while *P. hoplogaster* has a continuous distribution in similar heterogeneous macrobiomes and prefers damp microhabitats near lakes, rivers, and floodplains. *Philothamnus occidentalis* is found at forest edges and in wooded grasslands—in regions with summer and year round rainfall—that are frequently inundated (Bourquin 2004; Marais 2004; Schulze and Maharaj 2006). *Philothamnus natalensis* and *P. semivariegatus* are associated with forest‐savanna mosaics, with *P. natalensis* being more specialized in coastal forests, while *P. semivariegatus* extends into more arid macrobiomes such as arid savanna and Karoo scrub, where it also frequents microhabitats such as rocky outcrops. While these species are primarily sympatric within heterogeneous microbiomes, anurophagous specialists are closely associated with microhabitats conducive to anurophagy specialization, i.e., moist, damp, and marshy conditions near water bodies. In contrast, anuro‐saurophagous generalists are equally associated with closed, moist as well as open, dry environments.

Spatial density analysis of predation records relative to predator species occurrence supports our inferences of resource partitioning amongst the five *Philothamnus* species studied. Although our KDE analysis highlights broad‐scale spatial patterns of resource use consistent with anurophagous specialization in *P. hoplogaster* and *P. angolensis* relative to anuro‐saurophagous generalization in *P. occidentalis* , *P. natalensis* , and *P. semivariegatus* , we recognize that variation in snake size, sex, local prey availability, and bias reporting for predation events could influence dietary records. Nevertheless, the spatial clustering of frog‐dominated predation records amongst the five species across multiple localities supports the interpretation that their specialization is not restricted to a single population or region but may be characteristic of their broader ecological strategy.

Resource partitioning amongst *Philothamnus* species, encompassing dietary specialization and generalization as well as microhabitat use in sympatry and parapatry, likely evolved in response to biome shifts from closed forests to open habitat formations like savannas and arid biomes since the Early Miocene (Engelbrecht et al. 2021). Radiation into novel transitional and heterogeneous habitats may have resulted in ecological opportunity, particularly concerning dietary habits and microhabitat use, while character displacement may have mitigated ecological competition in sympatry and parapatry. In this context, both ecological opportunity and character displacement may help to maintain species boundaries within *Philothamnus*, particularly in areas of sympatry and along edges of parapatric distributions, e.g., cognate species *P. natalensis* and *P. occidentalis* (Engelbrecht et al. 2019). However, this hypothesis requires further testing and scrutiny to evaluate whether it meets the criteria for ecological character displacement in the context of adaptive radiation (Schluter 2000a, 2000b). Graded ecological specialization–generalization may include dietary habits, biome associations, and microhabitat use, highlighting the potential for multifaceted fine‐scaled adaptations that may facilitate the coexistence and diversity of *Philothamnus* species.

## 5 Conclusions

Morphological differences for inferred dietary adaptations between *Philothamnus* anurophagous specialists and anuro‐saurophagous generalists appear distinct—indicating functional divergence in feeding strategies both at the outset and throughout the feeding process. We suggest that inferred dietary ecomorphology is intertwined with overall ecological resource partitioning impacted by historical biome affiliation, as well as present‐day microhabitat use along a specialization‐generalization continuum. Future studies should utilize a more extensive species dataset—such as mechanistic habitat‐use data—to gain deeper insights into the dietary ecomorphology and resource partitioning amongst the African Green and Bush snakes. In addition, including juvenile specimens could help reveal potential ontogenetic shape changes across species, further enriching our understanding of morphological variation related to diet amongst *Philothamnus* species. While diet likely plays a significant role in shaping skull form, it is certainly not the sole driver. Other factors, such as arboreal lifestyle, active foraging, lack of prey constriction, and the need for cranial balance and maneuverability during arboreal locomotion may also contribute to head shape evolution. Integrating feeding performance experiments will be crucial for validating the functional relevance of these inferred dietary adaptations. This approach may uncover saurophagous specialists at the opposite end of the dietary continuum, particularly for the *P. semivariegatus* lineage that is associated with the arid southwestern regions of Africa.

## Author Contributions

**Hanlie M. Engelbrecht:** conceptualization (lead), data curation (lead), formal analysis (lead), funding acquisition (lead), methodology (lead), project administration (lead), writing – original draft (lead), writing – review and editing (lead). **Kimberley E. J. Chapelle:** conceptualization (supporting), formal analysis (equal), writing – review and editing (supporting). **Graham J. Alexander:** validation (supporting), writing – review and editing (supporting).

## Disclosure

All results presented in this study are based solely on the authors' academic research and interpretation of the data.

## Conflicts of Interest

The authors declare no conflicts of interest.

## Data Availability

The data that supports the findings of this study are available in the Dryad Digital Repository at https://doi.org/10.5061/dryad.2jm63xt0g.

## Appendix B

Detailed shape variation across PC1 min–max and PC2 min–max for the maxilla, compound, quadrate, and pterygoid in anurophagous and anuro‐saurophagous snakes.

### Maxilla

*PC1 min*: anuro‐saurophagous generalists display a more posteriorly positioned ectopterygoid process on the maxillae and a more anteriorly positioned palatine process. At PC1 min, the anteromost point of the maxillae is more posteriorly positioned and the posteromost point is more anteriorly positioned. The bar between the palatine and ectopterygoid processes is mediolaterally narrower. The posteromost point of the maxillae is dorsoventrally more dorsally positioned in anuro‐saurophagous generalists.

*PC1 max*: the palatine‐ectopterygoid facet interdistance is shorter in anurophagous specialists. The anteromost point of the maxillae is more anteriorly and dorsoventrally positioned more ventrally The posteromost point is more posteriorly and dorsoventrally positioned more ventrally The bar between the palatine and ectopterygoid processes is mediolaterally wider in anurophagous specialists.

*PC2 min*: anurophagous specialists have an anteroposteriorly longer base of the ectopterygoid process on the maxillae and an anteroposteriorly as well as dorsoventrally shorter and mediolaterally narrower palatine process. At PC2 min both the anteriormost and the posteromost tips of the maxillae are posteriorly positioned.

*PC2 max*: anuro‐saurophagous generalists bear both the anteromost and the posteromost tips of the maxillae anteriorly.

### Compound

*PC1 min*: the surangulars of anuro‐saurophagous generalists show an anterodorsal most point of the mandibular fossa that is more anteriorly positioned and a dorsalmost point of the surangular crest that is more posteriorly positioned.

*PC1 max*: the surangulars of anurophagous specialists (especially, *P. hoplogaster* ) show an anterodorsal most point of the mandibular fossa that is more posteriorly positioned, and a dorsal‐most point of the surangular crest that is more anteriorly positioned. *P. angolensis* and *P. occidentalis* show a somewhat intermediate shape relative to *P. hoplogaster* and *P. natalensis* + *P. semivariegatus* .

*PC2 min*: anurophagous specialists have the anteromost point of the compound more posteriorly and the posteromost point more anteriorly. The anterodorsalmost point of the mandibular fossa and a dorsalmost point of the surangular crests are more posteriorly positioned. The posteromost point and the intramandibular hinge are more anteriorly positioned, and the intramandibular hinge appears to be mediolaterally wider. The saddle‐shaped articular surface with the quadrate is deeper and more anteriorly positioned.

*PC2 max*: anuro‐saurophagous generalists have the anteromost point of the compound more anteriorly and the posteromost point more posteriorly. The anterodorsalmost point of the mandibular fossa and dorsalmost point of the surangular crest are more anteriorly positioned. The posteromost point and the intramandibular hinge are more posteriorly positioned and mediolaterally narrower. The saddle‐shape articular surface with the quadrate is more superficial and posteriorly positioned.

### Quadrate

*PC1 min*: anurophagous specialists show an anterodorsally oriented dorsal margin, with the anterodorsal most point being more dorsally positioned. The supratemporal condyle is continuously sloping and wider. The stylohyal process is dorsoventrally longer and more ventrally positioned. The ventral fossa (articular surface with the compound) and condyles are dorsoventrally more extensive and mediolaterally broader.

*PC1 max*: anuro‐saurophagous generalists have a horizontally oriented dorsal margin, a more ventrally positioned anterodorsal point, and a more dorsally positioned posterodorsal point. The stylohyal process is dorsoventrally shorter and more dorsally positioned. Generalists also show a dorsoventrally lower ventral fossa and condyles which are mediolaterally narrower.

*PC2 min*: anurophagous specialists have a posteroventrally sloping dorsal margin with an anterodorsal point that is more dorsally positioned and a posterodorsal point that is more ventrally located. The stylohyal process extends further medially and the ventral fossa extends further dorsally (deeper articulation with the compound).

*PC2 max*: anuro‐saurophagous generalists have a dorsal quadrate margin that is subhorizontal. The stylohyal process is dorsoventrally short and mediolaterally narrow, and the ventral fossae are dorsoventrally shortened.

### Pterygoid

*PC1 min*: the pterygoids of anurophagous specialists have a more anteriorly positioned tooth row, and a more posteriorly and ventrally positioned ectopterygoid process. The posteriormost flank of the pterygoid extends more laterally, i.e., is more outward‐oriented. Anuro‐saurophagous generalists also have these pterygoid characteristics.

*PC1 max*: in some instances, anuro‐saurophagous generalists also have a posteriorly positioned tooth row, an anteriorly positioned ectopterygoid process that is more dorsally positioned. The posteriormost flank of the pterygoid is also more medially extended, i.e., inward‐facing, and is also longer.

*PC2 min*: in anurophagous specialists, the posterior margin at the toothrow is mediolaterally narrower and the posterior part of the pterygoid is more laterally extended.

*PC2 max*: in *P. semivariegatus* the posterior margin at the toothrow is mediolaterally wider and the posterior part of the pterygoid is medially extended.

## Appendix C

Akaike Information Criterion corrected for small sample sizes (AIC_c_), associated log‐likelihood scores (InL), delta AIC_c_ and weighted AIC_c_ scores for fitting evolutionary models to historical diet transition within *Philothamnus*. Specific models tested were: Brownian motion (BM), Ornstein‐Uhlenbeck (OU), early‐burst (EB), jump normal (JN), normal inverse Gaussian (NIG), Brownian + jump processes (BMJN and BMNIG).

**Table:**

Model	AIC_c_	lnL	∆AIC_c_	Weight
BM	46.99746	−21.5	80.58365	3.173e‐18
OU	48.99747	−21.5	82.58366	1.167e‐18
EB	43.81139	−18.9	77.39758	1.561e‐17
JN	−10.85646	8.43	22.72973	1.160e‐05
NIG	47.18470	−20.6	80.77089	2.889e‐18
BMJN	−33.58619	20.8	0.00000	9.999e‐01
BMNIG	49.18469	−20.6	82.77088	1.063e‐18
